# Transfer of *in vivo *primed transgenic T cells supports allergic lung inflammation and FIZZ1 and Ym1 production in an IL-4Rα and STAT6 dependent manner

**DOI:** 10.1186/1471-2172-12-60

**Published:** 2011-10-20

**Authors:** Preeta Dasgupta, Svetlana P Chapoval, Elizabeth P Smith, Achsah D Keegan

**Affiliations:** 1 Center for Vascular and Inflammatory Diseases, and Department of Microbiology and Immunology, University of Maryland School of Medicine, 800 W. Baltimore St., Baltimore, MD 21201, USA; 2 Center for Vascular and Inflammatory Diseases, University of Maryland School of Medicine, 800 W. Baltimore St., Baltimore, MD 21201, USA

## Abstract

**Background:**

CD4+ T helper type 2 (T_H_2) cells, their cytokines IL-4, IL-5 and IL-13 and the transcription factor STAT6 are known to regulate various features of asthma including lung inflammation, mucus production and airway hyperreactivity and also drive alternative activation of macrophages (AAM). However, the precise roles played by the IL-4/IL-13 receptors and STAT6 in inducing AAM protein expression and modulating specific features of airway inflammation are still unclear. Since T_H_2 differentiation and activation plays a pivotal role in this disease, we explored the possibility of developing an asthma model in mice using T cells that were differentiated *in vivo*.

**Results:**

In this study, we monitored the activation and proliferation status of adoptively transferred allergen-specific naïve or *in vivo *primed CD4+ T cells. We found that both the naïve and *in vivo *primed T cells expressed similar levels of CD44 and IL-4. However, *in vivo *primed T cells underwent reduced proliferation in a lymphopenic environment when compared to naïve T cells. We then used these *in vivo *generated effector T cells in an asthma model. Although there was reduced inflammation in mice lacking IL-4Rα or STAT6, significant amounts of eosinophils were still present in the BAL and lung tissue. Moreover, specific AAM proteins YM1 and FIZZ1 were expressed by epithelial cells, while macrophages expressed only YM1 in RAG2^-/- ^mice. We further show that FIZZ1 and YM1 protein expression in the lung was completely dependent on signaling through the IL-4Rα and STAT6. Consistent with the enhanced inflammation and AAM protein expression, there was a significant increase in collagen deposition and smooth muscle thickening in RAG2^-/- ^mice compared to mice deficient in IL-4Rα or STAT6.

**Conclusions:**

These results establish that transfer of *in vivo *primed CD4+ T cells can induce allergic lung inflammation. Furthermore, while IL-4/IL-13 signaling through IL-4Rα and STAT6 is essential for AAM protein expression, lung inflammation and eosinophilia are only partially dependent on this pathway. Further studies are required to identify other proteins and signaling pathways involved in airway inflammation.

## Background

CD4+ T helper type 2 (T_H_2) cytokines such as IL-4, IL-5 and IL-13 play a critical role in inducing allergy and asthma. These cytokines act on multiple cells types to initiate and propagate the hallmark features of asthma such as pulmonary inflammation, periodic narrowing of airways and mucus hypersecretion [1-7]. Experiments with mice deficient in these cytokines and studies in asthma patients have confirmed these findings [8-10]. Also, the fact that T_H_2 cells are required in this disease setting has been demonstrated by using IL-4^-/- ^mice and adoptive transfer studies [3,6,8,11]. Apart from T_H_2 cells, IL-4 and IL-13 are also secreted by natural killer (NK) T cells, basophils, mast cells, macrophages and activated eosinophils (reviewed in [12]).

IL-4 and IL-13 share receptor chains and signaling proteins. Binding of either cytokine to the Type I or Type II receptor complex leads to the phosphorylation of signal transducer and activator of transcription factor (STAT) 6 [12-14]. Polymorphisms in the *Il4ra *and *Stat6 *genes have been linked to increased risk of asthma [15,16]. There is ample evidence that IL-4 signaling through IL-4Rα and STAT6 is important for T_H_2 differentiation and for IgE class-switching in B cells [13,14]. Furthermore, mucus hypersecretion, goblet cell hyperplasia and airway hyperresponsiveness (AHR) were completely abolished in IL-4Rα^-/- ^or STAT6^-/- ^mice [1,4,17]. We have previously shown that apart from T_H_2 cells, IL-4Rα expression on a population of CD11b+ cells contributed to the severity of lung inflammation and eosinophil recruitment [7]. Although these signaling molecules have been studied extensively, there are conflicting reports in the literature regarding the roles of IL-4Rα and STAT6 in modulating specific features of airway inflammation. Some studies have shown that there was no eosinophil recruitment in STAT6^-/- ^mice [6], while other groups including us contend that lung eosinophilia and inflammation are only partially dependent on STAT6 [1,18].

Recently it has been established that IL-4 and IL-13 can promote differentiation of alternatively activated macrophages (AAM) (reviewed in [19,20]). During Type II inflammation, AAMs as well as epithelial cells produce certain characteristic factors such as Arginase 1, chitinase- like mammalian proteins (eg. YM1) and found in inflammatory zone (FIZZ; also named as Resistin- like molecule, RELM) proteins. Four different sub-types of FIZZ proteins have been reported in the literature- FIZZ1-4. FIZZ1 was originally discovered in the bronchoalveolar lavage (BAL) fluid in a mouse model of asthma [21]. Elevated levels of FIZZ1 and YM1 mRNA or protein have since been detected in parasite infection models [20,22], allergic lung inflammation [21,23,24], allergic peritonitis [24], bleomycin-induced lung fibrosis [25] and hypoxia-induced pulmonary hypertension [26]. Interestingly, the promoter regions of both FIZZ1 and YM1 have functional binding sites for STAT6 [23,24], which explains how IL-4 and IL-13 can induce expression of these proteins. Our group has shown previously using *in vitro *studies, that FIZZ1, YM1 and Arginase 1 mRNA are preferentially upregulated by IL-4 and to a lesser extent by IL-13 [27]. Loss of STAT6 signaling results in a significant reduction in FIZZ1 and YM1 mRNA levels in different model systems [24,25]. However, the effect of IL-4Rα or STAT6 on FIZZ1/YM1 protein induction in an asthma model has not yet been studied.

It is well established that signaling through the IL-4 pathway is important for T_H_2 cell differentiation and cytokine production (reviewed in [28]). IL-4 signaling through the IL-4R/STAT6 axis upregulates GATA3, the T_H_2 master transcription factor. Adoptive transfer of wild type T_H_2 cells into mice is an effective tool to bypass the role of STAT6 and IL-4Rα in T_H_2 cell differentiation, while allowing the study of these signaling molecules in the effector phase of asthma. Most groups use *in vitro*-generated T_H_2 effectors for this purpose. However, this protocol involves several rounds of *in vitro *priming and expansion using large quantities of IL-4. Also, it is becoming evident that *in vivo *T_H_2 differentiation is more complex and that certain signals may be missing when cells are differentiated *in vitro *(reviewed in [29]). It is now known that TSLP, IL-25 and IL-1 family cytokines can be involved in induction or maintenance of the T_H_2 phenotype [29]. In order to avoid using *in vitro *T cell activation, we isolated Ovalbumin (OVA)-specific CD4+ T cells from either unimmunized DO11.10xRAG2^-/- ^mice to get naïve CD4+ T cells or DO11.10xRAG2^-/- ^mice immunized with OVA/Alum to get *in vivo*-primed CD4+ T cells. Previous dogma led to the assumption that it would be difficult to immunize TCR transgenic mice [30,31] as T cells in these mice undergo reduced proliferation. However, recent reports indicate that it is possible to immunize TCR transgenic mice and that T cell proliferation and cytokine production are independent of each other [32,33].

In this study, we found that *in vivo *primed CD4+ T cells underwent reduced proliferation in a lymphopenic environment when compared to naïve CD4+ T cells. Using this modified asthma model with *in vivo *generated effector T cells, we demonstrate that although there was a reduction in recruitment of inflammatory cells and eosinophils in absence of IL-4Rα or STAT6, significant amounts of eosinophils were still present both in the BAL and also the lung tissue of STAT6xRAG2^-/- ^and IL-4RαxRAG2^-/- ^mice. Interestingly, YM1 protein was expressed by both epithelial cells and macrophages, while FIZZ1 protein was only expressed by epithelial cells in RAG2^-/- ^mice. We further show that FIZZ1 and YM1 protein expression in the lung was completely dependent on signaling through the IL-4Rα and STAT6. Thus it appears that while IL-4/IL-13 signaling through IL-4Rα and STAT6 is essential for induction of AAM genes, lung inflammation and eosinophilia are only partially dependent on this pathway.

## Results

### Activation and proliferation status of naïve versus in vivo primed T cells

**Figure 1:**
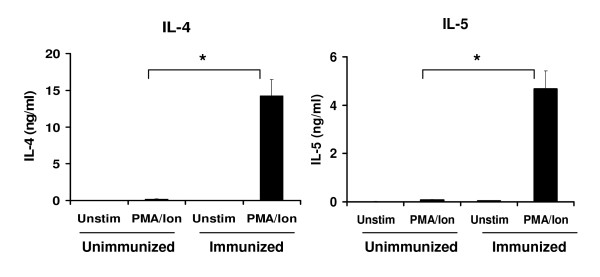
**Cytokine production by unimmunized and OVA/Alum immunized D011.10xRAG2^-/- ^mice**. Splenocytes isolated from either naïve (unimmunized) or OVA/Alum immunized D011.10xRAG2^-/- ^mice were cultured in media containing 20 U/ml IL-2 and were either stimulated with PMA/Ionomycin (PMA/Ion) or left untreated for 18 h. IL-4 and IL-5 secretion into the cell culture supernatant was quantitated using ELISA. Data represented as concentration of cytokine ± SEM in ng/ml. n = 3 for each group. * p < 0.05.

Since it has been suggested that T_H_2 differentiation *in vivo *is quite complex [29] and cannot necessarily be mimicked by *in vitro *priming of T cells, we tested the possibility of using either naïve or *in vivo *primed CD4+ DO11.10+ T cells. First, we determined if the *in vivo *primed T cells were capable of secreting more T_H_2 cytokines as a result of OVA/Alum immunization of D011.10xRAG2^-/- ^mice. Splenocytes isolated from either unimmunized (naïve) or immunized D011.10xRAG2^-/- ^mice were stimulated with PMA and Ionomycin and secretion of IL-4 into the cell culture supernatant was measured by ELISA. We found that, just one round of priming with OVA/Alum was sufficient to induce robust secretion of T_H_2 cytokines, IL-4 and IL-5 (Figure 1). The amount of IL-4 and IL-5 produced by cells isolated from immunized mice (14.2 ng/ml and 4.67 ng/ml respectively) was significantly higher than that of naïve cells (0.17 ng/ml and 0.07 ng/ml).

**Figure 2:**
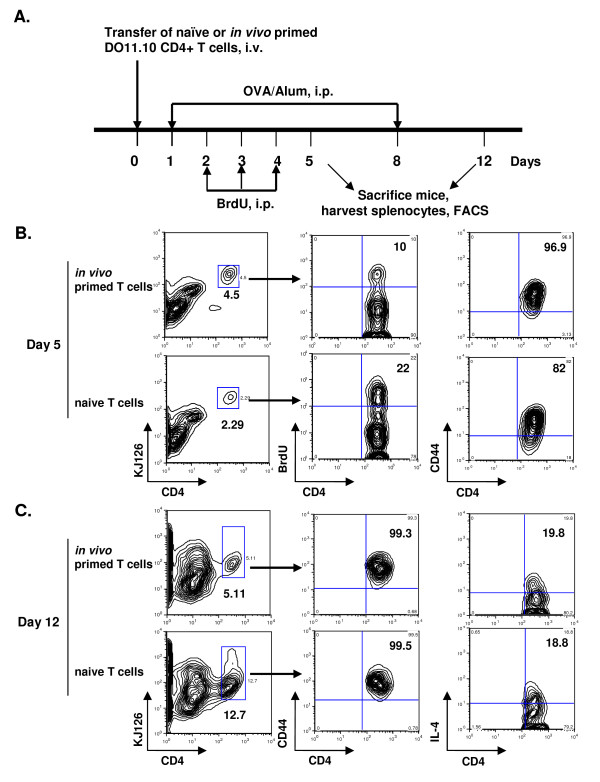
**Comparison of proliferation and activation status of naïve vs. *in vivo *primed T cells**. (A) Schematic representation of the protocol used in this experiment. Briefly, 1.5 × 10^6 ^naïve or *in vivo *primed CD4+ T cells were adoptively transferred into STAT6xRAG2^-/- ^mice and primed with OVA/alum i.p. on day 1. One group of mice was treated daily with BrdU (1 mg/mouse) i.p for 3 days before harvesting spleens on day 5. Splenocytes were pooled together and total cell counts were recorded. Cells were stained with anitbodies to CD4, KJ126, CD44 and BrdU and flow cytometry was performed. Another group of mice, that didn't receive BrdU were immunized with OVA/alum a second time on day 8. Four days later, splenocytes were harvested, counted and stimulated with PMA (50 ng/ml) and Ionomycin (1 μg/ml) for 6 h. (B) BrdU and CD44 expression in the CD4+ KJ126+ population in the naïve T cell or *in vivo *primed T cell transfer groups are shown. (C) CD44 expression in the CD4+ KJ126+ population in naïve vs. *in vivo *primed T cell transfer groups on day 12 is shown. IL-4 production by naïve and *in vivo *primed DO11.10 CD4 T cells was measured by intracellular cytokine staining (ICS).

Next we compared the ability of the naïve and *in vivo *primed CD4+ T cells to be activated upon OVA/Alum priming. Since differentiation of CD4+ T cells can often be accompanied by cell proliferation, we also monitored T cell proliferation using 5-bromo-2-deoxyuridine (BrdU) incorporation. In order to test this, we transferred naïve or *in vivo *primed T cells into STAT6xRAG2^-/- ^mice by tail vein injection, followed by immunization with OVA/Alum using a protocol shown in Figure 2A. One group of mice was injected with BrdU. Splenocytes were harvested on day 5 and analyzed by flow cytometry. We found that although the total number of splenocytes isolated from these two groups was similar (22.75 × 10^6 ^and 23 × 10^6 ^cells respectively; Table 1), the number of DO11.10+ CD4+ T cells in the *in vivo *primed transfer group was double that of the naïve transfer group- 4.5% vs. 2.29% (Figure 2B); 587 cells vs. 267 cells (Table 1). However the percentage of naïve DO11.10+ CD4+ T cells that were proliferating, as measured by BrdU incorporation was higher when compared to the *in vivo *primed DO11.10+ CD4+ T cells- 22% vs. 10% (Table 1). We also looked at cell surface activation markers such as CD44 (hyaluronic acid receptor) expressed by these cells. The majority of cells in both groups on day 5 were CD44hi, although expression of this protein was slightly higher in the group receiving *in vivo *primed T cells (Figure 2B and Table 1). Interestingly, although only 10% of the CD4+ T cells in the primed T cell transfer group and 22% of these cells in the naïve T cell transfer group were BrdU+, CD44hi expression by these cells in the two groups of mice was 97% and 82% respectively. This suggests that even cells that did not divide in both groups were expressing high levels of CD44 and were activated.

**Table 1:** Comparison of cells present in mice receiving naïve or *in vivo *primed CD4+DO11

	Total Splenocytes	# of CD4+ DO11.10+ lymphocytes	% BrdU+	% CD44+
**STAT6xRAG2 + primed CD4 T cells**	23 × 10^6 ^cells	587 cells	10%	96.9%
**STAT6xRAG2 + Naïve CD4 T cells**	22.75 × 10^6 ^cells	267 cells	22%	82%

Cell proliferation studies were conducted using the protocol mentioned in Figure 2 and materials and methods. Briefly, naïve or *in vivo *primed CD4+ T cells were adoptively transferred into STAT6xRAG2^-/- ^mice on day 0, followed by OVA/alum immunization of day 1. Mice were treated with BrdU i.p for 3 days. On day 5, splenocytes were harvested, single cell suspensions were prepared and total cell numbers were counted (column 1). Cells were stained with fluorochrome conjugated antibodies and analyzed by flow cytometry. Lymphocytes were gated based on forward and side scatter parameters. The CD4+ DO11.10+ population in each transfer group was gated based on double expression of CD4 and KJ126 by each cell (column 2). BrdU and CD44 expression in these gated cells was examined (columns 3 and 4 respectively). The numbers/percentages in columns 2-4 were determined by FACS Analysis. 20,000 events (splenocytes) were collected for each tube/analyte.

Another set of STAT6xRAG2^-/- ^mice that received either naïve or *in vivo *primed DO11.10 T cells were immunized twice with OVA/Alum within a span of 1 week (Figure 2A; similar to the antigen sensitization protocol used in the allergic lung inflammation experiments). The activation status of the adoptively transferred T cells and the amount of IL-4 produced by these cells was monitored. The number of spleen cells recovered at this stage (day 12) from the naïve T cell transfer group was twice as much as the number recovered from spleens of mice that had received *in vivo *primed T cells (Table 2). Moreover, the percentages of naïve DO11.10+ CD4+ T cells (12.7%) in the spleen were also higher when compared to that of the *in vivo *primed DO11.10+ CD4+ T cells (5.11%; Figure 2b). This result correlates well with the BrdU incorporation data and indicates that naïve DO11.10+ CD4+ T cells proliferate more in a lymphopenic environment, which leads to greater accumulation of these cells in spleens of mice at a later time. This accumulation of T cells may be due to homeostatic proliferation. However, the percentage of cells expressing CD44 (> 99%) and IL-4 (18.8% and 19.8%) was similar in both the naïve and *in vivo *primed groups on day 12 (Table 2 and Figure 2C).

**Table 2:** Comparison of cells present in mice receiving naïve or *in vivo *primed CD4+DO11

	Total Splenocytes	# of CD4+ DO11.10+ lymphocytes	% CD44+
**STAT6xRAG2 + primed CD4 T cells**	157 × 10^6 ^cells	328 cells	99.3%
**STAT6xRAG2 + Naïve CD4 T cells**	350 × 10^6 ^cells	629 cells	99.5%

T cell activation studies were conducted using the protocol mentioned in Figure 2 and materials and methods. Briefly, naïve or *in vivo *primed CD4+ T cells were adoptively transferred into STAT6xRAG2^-/- ^mice on day 0, followed by OVA/alum immunization of days 1 and 8. Four days later, splenocytes were harvested, single cell suspensions were prepared and cells were counted (column 1). Splenocytes were stimulated with PMA and Ionomycin for 6 h, followed by FACS staining and analysis. Lymphocytes were gated based on forward and side scatter parameters. The CD4+ DO11.10+ population in each transfer group was gated based on double expression of CD4 and KJ126 by each cell (column 2). CD44 expression in these gated cells was examined (column 3). The numbers/percentages in columns 2 and 3 were determined by FACS Analysis. 20,000 events (splenocytes) were collected for each tube/analyte.

### Effect of STAT6 and IL-4Rα on lung inflammation, eosinophilia and mucus production

Considering that naïve DO11.10+ CD4+ T cells were proliferating more in a lymphopenic environment and since we wanted to focus on the effector functions of IL-4 and IL-13 but not their role in priming naïve T cells, we chose the *in vivo *primed DO11.10+ CD4+ T cells for all further experiments.

**Figure 3:**
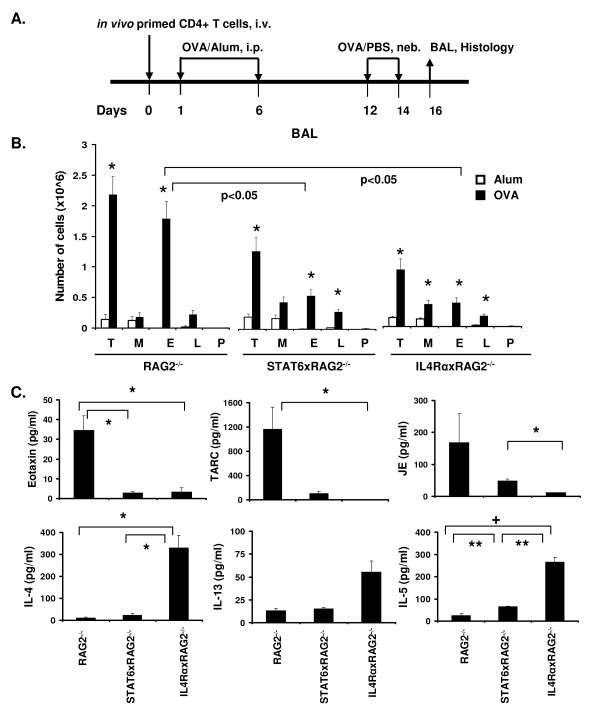
**Degree of BAL eosinopilia, cytokine and chemokine secretion in RAG2^-/- ^STAT6xRAG2^-/- ^or IL-4RαxRAG2^-/- ^mice**. The asthma protocol used in this study is depicted in (A). *In vivo *primed DO11.10+ CD4+ T cells were adoptively transferred into RAG2^-/-^, STAT6xRAG2^-/- ^or IL-4RαxRAG2^-/- ^mice. Mice were primed with either alum or 100 μg of Ova in alum i.p on d. 1 & 6 and then challenged with nebulized PBS or 1% Ova in PBS on d. 12 &14. BAL fluid was recovered 48 h after the last challenge and cells were analyzed by differential staining. Lung tissue was collected for histological analysis. (B) The total number of cells (T), macrophages (M), eosinophils (E), lymphocytes (L) and polymorphoneutrophils (P) present in the BAL in RAG2^-/-^, STAT6xRAG2^-/- ^and IL-4RαxRAG2^-/- ^mice are represented here in the form of bar graphs. Data represented as numbers ± SEM. * (p < 0.05), represents statistically significant differences between the OVA and Alum treated mice in each group. n = 5 for Ova treated mice, n = 3 for alum treated. (C) Chemokine and cytokine levels in BAL samples from OVA primed and challenged RAG2^-/-^, STAT6xRAG2^-/- ^and IL-4RαxRAG2^-/- ^mice were analyzed using a multiplex array system. Data are presented as mean chemokine or cytokine level in pg/ml ± SEM. * p < 0.05; ** p < 0.01; **+ **p < 0.0001. n = 4 for RAG2^-/- ^mice, n = 3 each for STAT6xRAG2^-/- ^or IL-4RαxRAG2^-/- ^mice. Representative data from one of three independent experiments is shown.

Several groups including ours have shown that IL-4 and IL-13 signaling through IL-4Rα and STAT6 plays an important role in inducing and exacerbating eosinophilic inflammation and mucus production in the lungs [1,5-7,16,18]. Since some of these studies were conducted using *in vitro *generated T_H_2 effectors, we examined whether similar responses would be observed using *in vivo *primed T cells. Furthermore, although similar studies have been conducted with STAT6^-/- ^mice or IL-4Rα^-/- ^mice alone [1,6,7], no head to head comparisons between mice deficient in STAT6 or IL-4Rα have been made. To tease out the precise roles played by these signaling molecules, we conducted allergic inflammation studies on RAG2^-/-^, STAT6xRAG2^-/- ^and IL-4RαxRAG2^-/- ^mice using our model of transferring *in vivo *primed T cells (Figure 3A).

The degree of airway inflammation, eosinophil recruitment and mucus production in the lungs was analyzed in the three groups of mice. As reported earlier [1,7], priming with alum alone did not induce eosinophilia and airway inflammation (Figure 3B) and served as a negative control. Upon enumerating the cellular composition in the BAL, we found that the total number of cells recovered from OVA treated RAG2^-/- ^mice was significantly higher (2.1 × 10^6 ^cells) than the number of cells recovered from OVA treated STAT6xRAG2^-/- ^and IL-4RαxRAG2^-/- ^mice (1.26 × 10^6 ^and 0.9 × 10^6 ^cells respectively). (Figure 3B). Among the different cell types (macrophages, eosinophils, lymphocytes and neutrophils) found in the BAL, a 2-3 fold reduction in the numbers and percentages of eosinophils was seen in STAT6xRAG2^-/- ^and IL-4RαxRAG2^-/- ^mice when compared to RAG2^-/- ^mice challenged with OVA (Figure 3B and additional file 1, Figure S1A). In each case, the numbers of eosinophils, macrophages and lymphocytes present in the OVA treated mice were much greater than the alum treated mice (Figure 3B).

H&E stained lung sections of OVA treated RAG2^-/- ^mice demonstrated severe lung inflammation (Additional file 1, Figure S1B, panel a) and most of the cellular infiltrate was composed of eosinophils (Additional file 1, Figure S1B, panel b). Multinucleated giant cells (MNGs) were also present in large numbers. In contrast, in absence of STAT6 and IL-4Rα only minor cuffing of the airways and blood vessels was observed (Additional file 1, Figure S1B, panels d & g respectively). Eosinophil recruitment into the lung although reduced, was not completely abolished in STAT6xRAG2^-/- ^and IL-4RαxRAG2^-/- ^mice (Additional file 1, Figure S1B, panels e & h respectively). PAS staining on the above lung sections indicated that mucus production by epithelial cells was fully dependent on STAT6 and IL-4Rα (Additional file 1, Figure S1B, panels c, f and i). This is not surprising as it known that mucus production is mainly driven by IL-13 mediated STAT6 activation [4,5,34].

The differences in eosinophil counts in the BAL (Figure 3B) in the three mouse strains were recapitulated in the lung tissue. The number of eosinophils recruited to the airways and blood vessels in OVA primed and challenged STAT6xRAG2^-/- ^and IL-4RαxRAG2^-/- ^mice was significantly reduced but not completely absent (Additional file 2, Figure S2A & B). A corresponding increase in mononuclear cell infiltration was observed in these mice. Surprisingly, the number of mononuclear cells in STAT6 deficient mice was higher than in IL-4Rα deficient mice. It is possible that this is linked to the presence of increased numbers of myeloid progenitor cells that have been reported in STAT6^-/- ^mice [35]. However, we found significantly higher eosinophils in the lung parenchyma in STAT6xRAG2^-/- ^and IL-4RαxRAG2^-/- ^mice, when compared to RAG2^-/- ^mice (Additional file 2, Figure S2C).

Taken together, these results suggest that *in vivo *primed CD4+ T cells can induce robust allergic lung inflammation in mice. In this model, STAT6 and IL-4Rα expression are only partially required for inducing pulmonary inflammation and eosinophilia.

### Chemokine and cytokine profile in the BAL in presence or absence of STAT6 and IL-4Rα

IL-4 and IL-13 signaling can induce production of many chemokines by different cell types. Eotaxin-1 (CCL11) and Eotaxin-2 (CCL24) are eosinophil chemoattractive proteins that are predominantly produced by epithelial cells in mice (reviewed in [36]), upon IL-4 or IL-13 stimulation [37,38]. Previous studies have shown that induction of eotaxin, eotaxin 2 and TARC mRNA in the lungs of OVA-challenged mice was STAT6 dependent [6,37]. We determined the quantities of eotaxin, TARC and mouse JE/CCL2 secreted into the BAL (Figure 3B, panel b). Using our model of *in vivo *primed T cell transfer and OVA-induced allergic lung inflammation, we further show that significantly elevated levels of eotaxin and TARC protein were found in RAG2^-/- ^mice when compared head to head with STAT6xRAG2^-/- ^and IL-4RαxRAG2^-/- ^mice. A similar trend is seen in the case of JE/CCL2 production. Since eotaxin plays an important role in eosinophil trafficking, the reduced amount of eotaxin found in the BAL of STAT6xRAG2^-/- ^and IL-4RαxRAG2^-/- ^mice could explain the lower numbers of eosinophils present around the airways in mice (Figures 3B and S2).

As T_H_2 cytokines have been implicated in allergic lung inflammation, we evaluated IL-4, IL-5 and IL-13 secretion into the lungs and analyzed the contribution of STAT6 and IL-4Rα head to head in this process, using our *in vivo *primed T cell model. Since we provided WT OVA-specific T cells to all three groups of mice, these cells would be able to produce T_H_2 cytokines. We found that upon priming and challenge with OVA, both RAG2^-/- ^and STAT6xRAG2^-/- ^mice secreted similar amounts of IL-4 and IL-13 into the BAL (Figure 3C, bottom left). However, significantly higher levels of IL-4 were present in the BAL of IL-4RαxRAG2^-/- ^mice when compared to the other two groups (Figure 3C). Although not significant, IL-13 secretion in these mice followed a similar trend. It is published that binding of IL-4 to the IL-4R complex induces internalization and uptake of the cytokine [39]. Thus, in mice deficient in IL-4Rα, absence of the IL-4R on cell surfaces may be preventing the internalization of IL-4 and IL-13, thus increasing the concentration of these cytokines in the BAL. Similar results were obtained by other groups when antibodies against the IL-4Rα chain or IL-13Rα1 were used [34,40].

In case of IL-5, increasing amounts of this cytokine was detected in the three mouse strains, with the lowest quantity of IL-5 present in the BAL of RAG2^-/- ^mice, intermediate levels in STAT6xRAG2^-/- ^mice and the highest in IL-4RαxRAG2^-/- ^mice (Figure 3C, bottom right). Studies have shown that when *in vitro *generated T_H_2 effectors were adoptively transferred into STAT6^-/- ^mice, there was a dramatic increase in IL-5 secretion in the BAL [6]. The authors speculated that this difference was due to decreased consumption of IL-5 by eosinophils. In our model also, we find that there is a correlation between the degree of eosinophilic inflammation in mice and the amount of IL-5 present in the BAL. Thus the lower levels of IL-5 found in the BAL fluid in RAG2^-/- ^mice may be explained by increased consumption of this cytokine by eosinophils recruited into the lungs (seen in Figure 3B and additional file 2 Figure S2).

### Migration of T_H_2 cells into the lungs is independent of STAT6 expression

**Figure 4:**
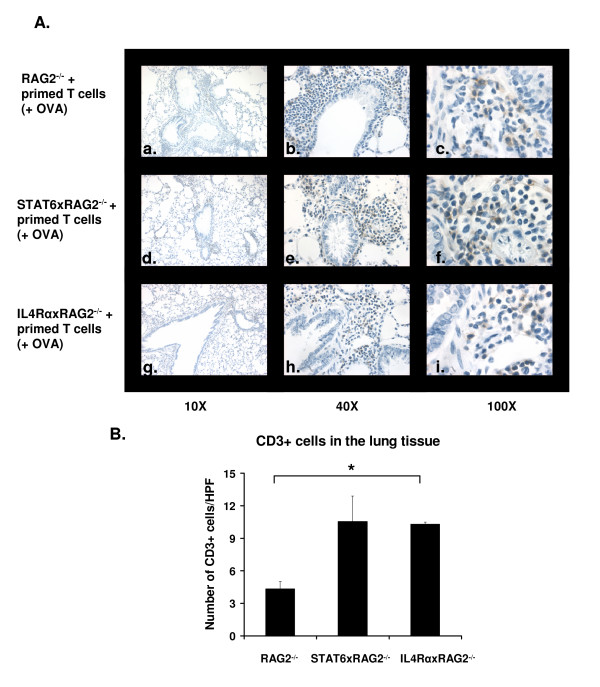
**CD3+ T cells migrate into the lung in absence of STAT6**. Allergic lung disease was induced in RAG2^-/-^, STAT6xRAG2^-/- ^or IL-4RαxRAG2^-/- ^mice as described above. Images (10×, 40× and 100× magnifications) of representative lung sections stained with antibodies to CD3 are shown in (A). CD3+ cells appear brown. Panels a-c: RAG2^-/- ^lung sections; d-f: STAT6xRAG2^-/- ^sections and g-i: IL-4RαxRAG2^-/- ^sections. n = 5 for each mouse strain. (B) Graphical representation of the immunohistochemistry data shown above. Number of CD3+ cells in each lung section was counted and graphed. Data represented as cell counts ± SEM. HPF: high power field; 100×. * p < 0.05.

Previous studies have shown that STAT6 expression was necessary for T_H_2 cell trafficking into the lung upon inhalation of Ovalbumin. Mathew *et. al*. reported that in the absence of STAT6, less antigen specific T_H_2 cells migrated into the lungs [6]. To check if this was true in our studies, lung sections were stained with antibodies to CD3 to identify T cells. Since all mice were on a RAG deficient background, the only CD3+ T cells present in the lungs were the OVA-specific T cells that we adoptively transferred. As evident from Figure 4A, absence of STAT6 or IL-4Rα did not block migration of antigen specific T cells into the lungs of mice. When the CD3+ cells in these mice were quantified, we found that significantly greater numbers of T cells were recruited in the lungs of IL-4RαxRAG2^-/- ^mice when compared to RAG2^-/- ^mice and a similar trend was seen in STAT6xRAG2^-/- ^(Figure 4B). Thus when the T cells express STAT6 or IL-4Rα themselves, deficiency of those proteins in lung resident cells does not affect T cell trafficking.

### Effect of STAT6 and IL-4Rα on FIZZ1 and Ym1 protein expression

**Figure 5:**
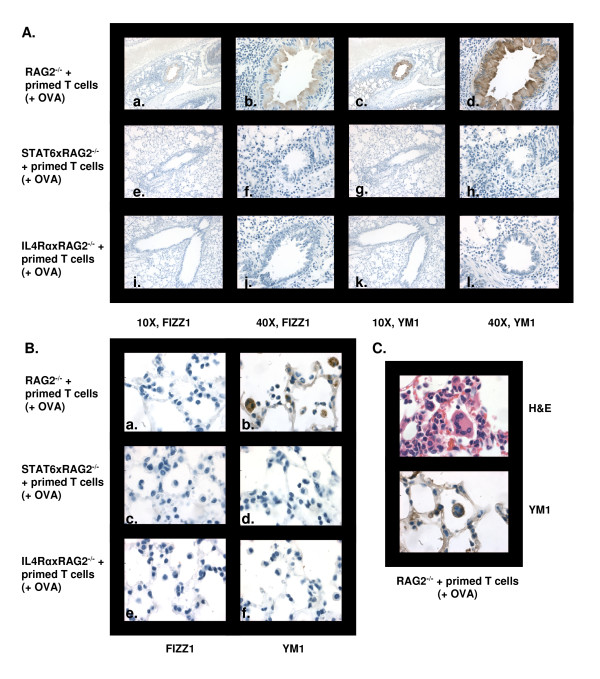
**FIZZ1 and YM1 expression in the lung is dependent on STAT6 and IL-4Rα**. Allergic lung inflammation was induced in RAG2^-/-^, STAT6xRAG2^-/- ^or IL-4RαxRAG2^-/- ^mice as mentioned in Figure 3 and materials and methods. FIZZ1 and YM1 expression was analyzed in serial sections of mouse lungs by immunohistochemistry. Photomicrographs of FIZZ1 and YM1 expression in epithelial cells (A) and macrophages (B) in representative lung sections are shown. (C) YM1 expression in multinucleate giant cells (MNG) in RAG2^-/- ^mice. Images in (B) and (C) are of 100× magnification.

Liu *et. al *reported that induction of FIZZ1 transcripts was STAT6 dependent in a bleomycin-induced lung fibrosis model [25]. YM1 mRNA was also upregulated in a STAT6 dependent manner in a mouse model of allergic peritonitis [24]. However, the expression patterns of these AAM proteins by epithelial cells and macrophages have not been studied in allergic lung inflammation. Moreover, we have observed a disconnect between the amounts FIZZ1 mRNA and protein induced by IL-4 stimulated macrophages *in vitro *[27]. Thus, we examined the expression profile of FIZZ1 and YM1 protein in our model and investigated the role of STAT6 and IL-4Rα in upregulation of these proteins. Serial lung sections from OVA sensitized and challenged RAG2^-/-^, STAT6xRAG2^-/- ^and IL-4RαxRAG2^-/- ^mice were stained with antibodies against both YM1 and FIZZ1 by immunohistochemistry (Figure 5A). Lung epithelial cells in RAG2^-/- ^mice stained strongly for FIZZ1 (Figure 5A, panel a & b) and YM1 (panel c & d). However, macrophages from these mice were positive for only YM1 but not FIZZ1 (Figure 5B, panel a & b). Multinucleated giant cells present in the lungs of RAG2^-/- ^mice also expressed YM1 (Figure 5C). In comparison, no FIZZ1 or YM1 protein was produced by epithelial cells (Figure 5A, panel e-h and i-l) or macrophages (Figure 5B, panel c, d and e, f) in mice deficient in IL-4Rα or STAT6.

**Figure 6:**
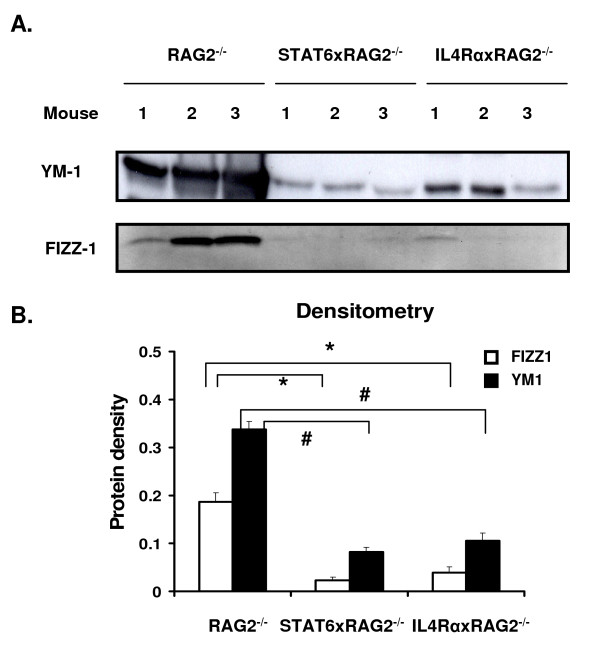
**Presence of FIZZ1 and YM1 protein in BAL fluid**. BAL fluid samples from RAG2^-/-^, STAT6xRAG2^-/- ^or IL-4RαxRAG2^-/- ^mice treated as described in Figure 4 were collected. FIZZ1 and YM1 protein secreted into the BAL fluid in the three groups of mice was detected by western blotting (A). Equal amounts of total protein were loaded into every well. Each lane represents an individual mouse. Densitometry analysis was performed on the autoradiograms from each blot and the values are represented on a graph (B). White bars represent densitometry values for FIZZ1, black bars represent YM1. * p < 0.01; # p < 0.001. n = 3 for each group.

To quantify the amount of FIZZ1 and YM1 protein that was produced by each mouse strain, we analyzed the expression pattern of these proteins secreted into BAL fluid by western blotting. Total protein present in the BAL fluid samples from RAG2^-/-^, STAT6xRAG2^-/- ^and IL-4RαxRAG2^-/- ^mice was first quantitated; more total protein was recovered from RAG2^-/- ^BAL when compared to mice lacking STAT6 or IL-4Rα (data not shown). Generally, the amount of total protein present in BAL correlates with the degree of inflammation seen in mice. In order to compare the quantities of FIZZ1 and YM1 present in the different mouse strains, equal amounts of total BAL protein from RAG2^-/-^, STAT6xRAG2^-/- ^and IL-4RαxRAG2^-/- ^mice were used. The BAL protein samples were resolved by polyacrylamide gel electrophoresis, transferred onto a membrane and probed with antibodies to YM1 or FIZZ1. Similar to the immunohistochemistry study, large amounts of FIZZ1 and YM1 were secreted into the BAL in RAG2^-/- ^mice, but this was greatly reduced in the absence of STAT6 and IL-4Rα (Figure 6A). Densitometry analysis of the blots revealed that the differences seen were significant (Figure 6B). These results demonstrate that STAT6 activation through IL-4Rα signaling is required for expression of FIZZ1 protein in lung epithelial cells and YM1 protein in macrophages and epithelial cells during allergic lung inflammation.

### Effect of STAT6 and IL-4Rα on airway remodeling

**Figure 7:**
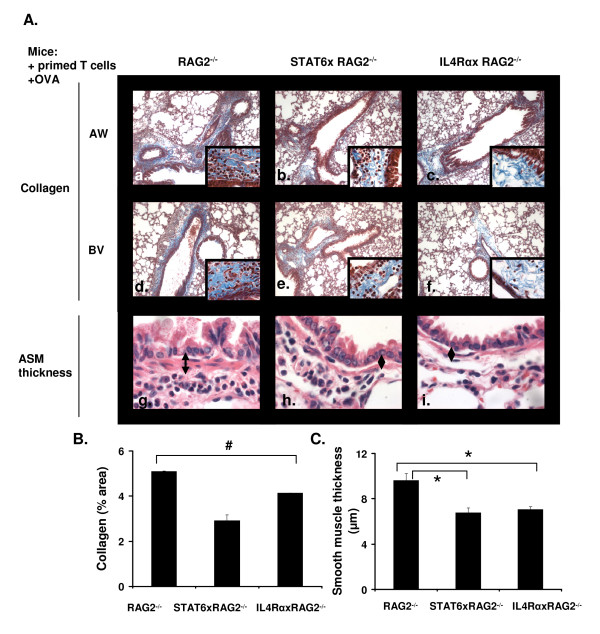
**Reduced airway remodeling in mice deficient in STAT6 and IL-4Rα**. RAG2^-/-^, STAT6xRAG2^-/- ^or IL-4RαxRAG2^-/- ^mice were subjected to the asthma protocol described in Figure 3. (A) Paraffin embedded lung sections from each group of mice were stained with Masson's Trichrome. Keratin and muscle fibers are stained in red, collagen in blue, cytoplasm in light red/pink and nuclei in black. Photomicrographs of collagen deposition around the airways (panels a-c) or blood vessels (panels d-f) were collected at 10× (panels a-f) and 100× (inset) magnification. Photomicrographs (100×) of the airway smooth muscle (ASM) layer in H&E stained lung sections from each mouse group is shown in panels g-i. Arrows depict the thickness of the ASM layer (transverse section). (B) The amount of collagen present in the lung was quantified using NIH Image J software. Data is represented as area of collagen (blue stain) ± SEM. # p < 0.001. n = 20 airways/blood vessels per group. (C) The distance between the innermost aspect and outermost aspect of the smooth muscle was measured at 3 different positions around each airway, using NIH Image J software. Data is represented as airway smooth muscle thickness in μm ± SEM. * p < 0.01. An average of 30 airways was used for each group.

One characteristic feature of asthma is airway remodeling, which involves an increase in airway smooth muscle mass and enhanced collagen deposition. It has been reported that both eosinophils and AAM products such as FIZZ1 and YM1 can cause lung fibrosis and smooth muscle thickening [26,41-44]. Thus, we analyzed the amount of collagen deposition and airway smooth muscle thickness in RAG2^-/-^, STAT6xRAG2^-/- ^and IL-4RαxRAG2^-/- ^mice. Masson's Trichrome staining of representative lung sections from each mouse strain revealed that greater quantities of collagen (shown in blue) was present around the airways (Figure 7A, panel a) and blood vessels (panel d) in RAG2^-/- ^mice, when compared with mice lacking STAT6 and IL-4Rα (Figure 7A, panels b&c, e&f). Quantification of the collagen staining using image analysis software showed that the differences were significant (Figure 7B). Furthermore, the thickness of the smooth muscle layer around the airways (the transverse diameter) was also significantly reduced in absence of STAT6 and IL-4Rα (Figure 7A and 7C). The airway smooth muscle layer was identified by H&E staining of lung sections (Figure 7A, panels g-i) and the diameter of the muscle layer was measured at three different points in each airway examined, using Image J software [45,46] (Figure 7C).

## Discussion

Although research on the cytokines IL-4 and IL-13 over the past decade has substantially increased our understanding of their contribution to the pathophysiology of asthma, the extent to which the signaling pathways they activate play a role in airway inflammation and alternative activation of macrophages have not yet been determined. In this study, we used STAT6 and IL-4Rα deficient mice on a RAG2^-/- ^background to examine the role of the IL-4/IL-13 pathway in inducing the aforementioned features of allergic lung disease. Since T_H_2 cells are indispensable in this disease setting, we provided T cells exogenously. Previously, most groups used *in vitro *generated T_H_2 effectors for this purpose [6,7]. Here, we developed a model wherein *in vivo *primed ovalbumin-specific CD4+ T cells were adoptively transferred into various recipient mice, followed by immunization and challenge with OVA. We examined whether naïve CD4+ T cells or *in vivo *primed CD4+ T cells isolated from DO11.10x RAG2^-/- ^would be more suitable for this asthma model. We found that *in vivo *primed T cells proliferated less when compared to naïve T cells as suggested by the following results: i. lower levels of BrdU incorporation in cells; ii. the number of CD4+DO11.10+ cells recovered from lymphopenic mice was half of that recovered from the naïve T cell transfer group. This increased proliferation seen in the naïve T cell transfer group may be due to homeostatic proliferation. It has been demonstrated, that naïve T cells from TCR transgenic mice undergo slow homeostatic proliferation in lymphopenic mice, which is dependent on IL-7 [47,48].

It has been proposed that entry into the cell cycle (i.e. cell proliferation) and clonal expansion is necessary for T cell differentiation [30,31,49]. Also, several groups have shown that TCR transgenic mice that have high frequency of antigen-specific T cells show only weak proliferation upon TCR ligation and that the T cells become anergic or die of apoptosis [50,51]. However, a study conducted by Laouar *et. al*. [32] and our studies have shown that when T cells from TCR transgenic mice were activated *in vivo *with specific peptide/antigen, these cells express cell surface activation markers such as CD44 and secrete effector cytokines, in spite of proliferating less (BrdU^- ^cells were expressing high levels of CD44). Why these transgenic T cells showed reduced proliferation is not known exactly, but it is hypothesized that at high cell frequency, there may be increased competition for growth factors, limited access to peptide/MHC complexes and also limited lymphoid space for expansion. The other difference between our study and the ones where transgenic T cells became anergic/apoptotic is the method of immunization: we used ovalbumin complexed with an adjuvant (alum) instead of using the antigen alone as was done previously. Thus, our results clearly show that *in vivo *primed CD4+ T cells from DO11.10 transgenic mice can be used to induce the hallmark features of asthma in mice. This effect is not restricted to one transgenic mouse strain; similar results were obtained when OT-II mice were used (data not shown).

In mice that lack STAT6 or IL-4Rα, T_H_2 cell differentiation is impaired but they have normal T_H_1 cell differentiation. In order to track the exogenous *in vivo *primed T cells that we were transferring into these mice and to prevent interference of T_H_1 cells, we used STAT6 or IL-4Rα deficient mice on a RAG2^-/- ^background for our asthma experiments. RAG2^-/- ^mice were used as controls. In this study, we tested the ability of *in vivo *primed CD4+ T cells as opposed to *in vitro *generated T_H_2 effectors to support allergic lung inflammation. We found that in the absence of STAT6 and IL-4Rα, mice developed less pulmonary inflammation, reduced perivascular and peribronchial cuffing and decreased eosinophilia than our control mice. Mucus production in these mice was abrogated. This was expected since it has been conclusively shown that mucus production is dependent on STAT6 activation by IL-13 signaling [4,5,34]. However, both STAT6xRAG2^-/- ^and IL-4RαxRAG2^-/- ^mice that were primed and challenged with OVA were able to recruit significantly higher numbers of eosinophils when compared to alum primed mice. Several studies have shown the importance of these signaling molecules in asthma, but the roles of IL-4Rα and STAT6 in modulating specific features of airway inflammation were unclear. Here we show that STAT6 and IL-4Rα are only partially required for eosinophil recruitment to the lung. Our data matches with what was observed by Kuperman *et. al*. [1] but is in apparent contradiction to that shown by Mathew *et. al*. [6]. Furthermore, in contrast to the latter's finding, we observe that there is no defect in T cell recruitment into the lung in absence of STAT6 or IL-4Rα. Differences in the experimental set up may explain these discrepancies: we and Kuperman *et. al*. used two priming steps with OVA/Alum in the asthma protocol, which was omitted by the other group. Recently it was demonstrated that alum stimulates the innate immune system by activating the Nalp3 inflammasome, leading to secretion of pro-inflammatory cytokines such as IL-1β, IL-18 and IL-33 [52]. These cytokines play important roles in initiation and amplification of T_H_2 responses including T_H_2 cell proliferation and eosinophilia [52,53]. Alum also induces production of IL-4 and IL-5 in T cells [54].

Eosinophil migration and recruitment into the lungs depend on several factors. IL-5 controls differentiation, activation and survival of eosinophils in the bone marrow and is essential for their mobilization into the lungs (reviewed in [36,55]). Eosinophil migration into the lung is mediated by adhesion to the vascular endothelium via VCAM-1 [56]. Chemokines produced by airway epithelial cells and macrophages recruit eosinophils into the airways [36]. Eotaxin-1 (CCL11) and Eotaxin-2 (CCL24) are eosinophil chemoattractive proteins, mainly produced by airway epithelial cells [36] upon IL-4 or IL-13 stimulation [37,38]. This was further substantiated by Munitz *et. al*. when they demonstrated that eotaxin secretion in mice is completely dependent on the IL-13Rα1 chain and thus the Type II IL-4/IL-13 receptor (the only receptor expressed on epithelial cells). In our studies, we show that eotaxin secretion in the BAL is markedly reduced in the absence of STAT6 or IL-4Rα. This might explain the lower levels of eosinophils seen around the airways and blood vessels in STAT6xRAG2^-/- ^and IL-4RαxRAG2^-/- ^mice in comparison to RAG2^-/- ^mice. However, in absence of eotaxin, IL-5 may play a major role in recruitment of eosinophils in STAT6xRAG2^-/- ^and IL-4RαxRAG2^-/- ^mice. The higher levels of IL-5 found in the BAL in these mice points to this direction. Other chemokines such as RANTES, Monocyte Chemoattractant Proteins (MCP) or Macrophage Inflammatory Protein (MIP)-1α could also play a role in this process. Interestingly, we found that greater numbers of eosinophils were present in the lung parenchyma in mice deficient in STAT6 and IL-4Rα. It is possible that in absence of eotaxin, eosinophils are unable to reach the airways and remain in the parenchyma.

In previous studies using WT and STAT6^-/- ^mice, T_H_2 cytokine production was higher in WT mice in comparison to mice lacking STAT6 [1]. This is because STAT6 is required for T_H_2 cell differentiation. Since we provided WT effector T cells to all the groups of mice, they were capable of producing T_H_2 cytokines. When we measured the amounts of IL-4 and IL-13 in the BAL in allergen challenged mice, we found that significantly higher amounts of these cytokines were present in IL-4RαxRAG2^-/- ^mice than RAG2^-/- ^and STAT6xRAG2^-/- ^mice. Studies have shown that binding of IL-4 to the IL-4R complex induces internalization and uptake of this cytokine [39], analogous to that observed with binding of IL-2 to the IL2R complex [57,58]. Moreover, other groups have found that IL-4 concentration in the BAL was significantly increased when antibodies against the IL-4Rα chain were injected into mice, compared to control mice [40]. Similarly, more IL-13 was found in the BAL in IL-13Rα1^-/- ^mice [34]. Therefore, based on our findings and published literature we hypothesize that the absence of IL-4Rα on cell surfaces may be preventing the internalization of IL-4 and IL-13, thus increasing the concentration of these cytokines in the BAL in IL-4RαxRAG2^-/- ^mice.

Our data also demonstrated that more IL-5 was secreted into the BAL when mice lacked STAT6 or the IL-4Rα chain. The higher concentration of IL-5 found in STAT6xRAG2^-/- ^mice in this model are consistent with the results reported by Mathew *et. al*. [6]. They had observed that when *in vitro *generated T_H_2 effectors were adoptively transferred into STAT6^-/- ^mice, there was a dramatic increase in IL-5 secretion in the BAL [6]. The authors speculated that this difference was due to decreased consumption of IL-5 by eosinophils. In our model, since the STAT6xRAG2^-/- ^and IL-4RαxRAG2^-/-^mice have significantly lower levels of eosinophils in both the BAL and lung tissue (Figure 3 and additional file 2, Figure S2), it is possible that the enhanced cytokine level in the BAL in these mice is due to reduced consumption. We did not see any significant differences in IFNγ levels in the three strains of mice. IL-17 is another cytokine that has been implicated in asthma in humans and mice (reviewed in [59]). In our asthma model, IL-17 levels in the BAL were below detection limits in all 3 mouse groups.

As our understanding of the roles of IL-4 and IL-13 increases, it is becoming clear that in addition to their action on T cells, B cells, eosinophils, epithelial cells, these cytokines can also stimulate macrophages such that they become alternatively activated. Instead of expressing iNOS like the classically activated macrophages, these cells produce proteins such as Arginase, FIZZ and YM1/2 among others (reviewed in [19,20]). It has now been established that IL-4 and IL-13 can also induce expression of the same group of proteins in airway and alveolar epithelial cells. As mentioned earlier, copious amounts of FIZZ1 and YM1 have been detected in the BAL of allergen challenged mice [21]. In addition, upregulated levels of FIZZ1 and YM1 mRNA have also been found in parasite infection models [20], allergic lung inflammation and allergic peritonitis [21,23,24], bleomycin-induced lung fibrosis [25] and hypoxia-induced pulmonary hypertension [60]. Stutz *et. al*. [23] demonstrated using the BMnot cell line that the FIZZ1 promoter contains functional binding sites for STAT6 and C/EBP. They further showed that STAT6 and C/EBP cooperated together to get optimal FIZZ1 expression. Welch *et. al*. [24] showed similar results for the YM1 promoter region using epithelial and macrophage cell lines: YM1 has several STAT6 binding sites in its promoter region. Additional studies have indicated that induction of FIZZ1 or YM1 transcripts are STAT6 dependent in bleomycin-induced lung fibrosis and allergic peritonitis model systems respectively [24,25].

In this article, we show that FIZZ1 and YM1 protein expression is induced upon allergen challenge in RAG2^-/- ^mice. We demonstrate for the first time in an allergic lung inflammation model that expression of these AAM proteins is completely dependent on signaling through IL-4Rα and STAT6. Furthermore, epithelial cells in control mice expressed both FIZZ1 and YM1 but macrophages expressed only YM1. This was surprising to us since we have shown previously that IL-4 induces a robust increase in FIZZ1 transcript levels in bone marrow macrophages (BMMs) within the first 6 hours of stimulation [27]. We failed to see FIZZ1 protein even when macrophages were stimulated with IL-4 *in vitro *for 48 hours. However, macrophages infected with nematodes or *Francisella tularensis *have been reported to express FIZZ1 protein [61]. The absence of this protein in macrophages in our studies could be due to decreased mRNA stability or posttranslational modification of the FIZZ1 mRNA, leading to its degradation. More studies are required to elucidate the molecular basis of this finding.

The functions of the AAM proteins in allergic lung inflammation and other diseases are still under debate and may differ depending on the disease setting and signaling pathways involved. FIZZ1 has been shown to have a protective effect in parasite infection studies. Two groups have shown independently that FIZZ1^-/- ^mice display significantly higher inflammation in the lung and liver, characterized by increased granuloma formation and extensive fibrosis when infected with *Schistosoma mansoni *or *Nippostrongylus brasiliensis *[20,22]. It was demonstrated that FIZZ1 is required for worm expulsion and also for suppressing excessive T_H_2 inflammation. On the other hand, FIZZ1 has been implicated in exacerbating pulmonary inflammation, vascular remodeling, collagen and extracellular matrix deposition leading to fibrosis in hypoxia-induced pulmonary hypertension and bleomycin-induced lung fibrosis models [26,43]. YM1 which is a secreted chitinase-like lectin binds to chitin but does not degrade it. It is postulated that YM1 is involved in worm egg degradation and tissue repair [62] and may also be involved in tissue remodeling [44]. More importantly, YM1 can act as an eosinophil chemoattractant [63,64].

The reduced expression of FIZZ1 and YM1 in the absence of STAT6 or IL-4Rα may be functionally linked to the reduced inflammation and eosinophilia seen in STAT6xRAG2^-/- ^and IL-4RαxRAG2^-/- ^mice. Moreover, we found that there was a significant decrease in collagen deposition and airway smooth muscle thickness in mice lacking STAT6 and IL-4Rα. Enhanced inflammation and eosinophilia as well as AAM products can contribute to airway remodeling. Studies have shown that depletion of the eosinophil lineage protected both humans and mice from deposition of collagen, extracellular matrix proteins and increases in smooth muscle mass [41,42,65,66]. It was recently shown that another chitinase-like protein BRP-39, was highly upregulated in alveolar macrophages and epithelial cells upon OVA sensitization and challenge [67]. In the absence of BRP39 there were reduced antigen-specific T_H_2 responses including IL-13 induced tissue inflammation and fibrosis. Based on these data and our earlier finding that IL-4 dramatically increases AAM gene expression in macrophages [27], we are inclined to think that elevated expression of AAM proteins including FIZZ1 and YM1 increases the severity of lung pathology.

## Conclusions

In summary, our data demonstrates that *in vivo *primed CD4+ T cells are able to support allergic lung inflammation. Moreover, STAT6 and IL-4Rα play a major role in a range of T_H_2 responses but the extent to which these signaling proteins control various aspects of allergic lung disease is variable. Our study establishes that STAT6 and IL-4Rα are necessary for FIZZ1 and YM1 protein induction but are only partially responsible for the recruitment of eosinophils and pulmonary inflammation. Further research is required to tease out the other pathways that are contributing to the severity of allergic lung inflammation.

## Methods

### Mice

Mice deficient in RAG2 (RAG2^-/-^) on a BALB/c background and DO11.10xRAG2^-/- ^transgenic mice containing T Cell Receptors (TCRs) specific for OVA peptide 323-339, were purchased from Taconic (Germantown, NY) or bred in the animal care facility at the University of Maryland, Baltimore (UMB). STAT6xRAG2^-/- ^mice were generated by crossing STAT6^-/- ^mice and RAG2^-/- ^mice [18]. The IL-4RαxRAG2^-/- ^mice were bred at Taconic under contract and then maintained at UMB. Both the STAT6xRAG2^-/- ^and IL-4RαxRAG2^-/- ^mice were on a BALB/c background. All experimental procedures mentioned here were performed in accordance to the guidelines issued by the Institutional Animal Care and Use Committee at the University of Maryland, Baltimore.

### Generation and adoptive transfer of naïve or in vivo primed CD4 T cells

DO11.10xRAG2^-/- ^mice were either used directly or immunized with 100 μg of chicken egg ovalbumin (OVA; Sigma-Aldrich, St. Louis, MO) adsorbed to aluminum hydroxide (alum; Sigma-Aldrich, St. Louis, MO) intraperitoneally (i.p). LN cells and splenocytes were harvested 10 days later to isolate naïve or *in vivo *primed T cells. These cells were treated with CD4 T cell negative selection enrichment cocktail and CD4+ T cells were purified either by using column separation (R&D Systems, Minneapolis, MN) or column-free immunomagnetic separation (Stem Cell Technologies, Vancouver, Canada). These cells were routinely > 90% pure. *In vivo *primed CD4+ T cells were injected intravenously (i.v.) via the tail vein in recipient mice (5 × 10^6 ^cells/mouse).

### In vivo proliferation assay and measurement of T cell activation

RAG2^-/- ^mice were adoptively transferred with ~2 × 10^6 ^naïve or *in vivo *primed CD4+ T cells from DO11.10xRAG2^-/- ^mice on day 0 and immunized with OVA/alum on day 1. Mice were treated daily with BrdU diluted in PBS (1 mg/mouse) i.p for 3 days. Splenocytes were isolated from two mice each for the naïve or *in vivo *primed groups, pooled together and stained with antibodies for CD4, KJ126, CD44 and BrdU. The cells were then analyzed by flow cytometry. A BrdU staining kit (BD Biosciences, San Jose, CA) was used for intracellular staining for BrdU. Another group of 4 mice that didn't receive BrdU, were immunized with OVA/alum a second time on day 8. 4 days later, splenocytes were harvested on day 12, counted and stimulated with PMA (50 ng/ml) and Ionomycin (1 μg/ml) for 6 h in the presence of Brefeldin A. CD44 expression and IL-4 production was monitored using flow cytometry.

### Antigen sensitization and challenge

Mice were sensitized and challenged with chicken egg ovalbumin using a modified protocol described by Wang *et al*. [68]. Each mouse was immunized with either 100 μg of OVA/alum or alum alone on day 1 and again on day 6. After the last sensitization step, mice were challenged with aerosolized PBS or 1% OVA in PBS for 40 minutes each day on days 12 and 14.

### Evaluation of airway inflammation

Mice were anaesthetized 48 hours after the last OVA challenge. Bronchial lavage was performed on each mouse by inserting a 1 mm tube into the trachea and flushing the lungs with 1 ml of PBS. The samples were centrifuged and the supernatant was used for cytokine analysis. The cellular component of the bronchoalveolar lavage (BAL) was resuspended in PBS. Total cell counts were determined. Cytospin preparations of the cells were stained with Diff-Quick (Dade Behring, Newark, DE) and differential cell counts were performed based on light microscopic analysis.

### Lung histology and Immunohistochemistry

Lung histology sections were prepared as described [7]. Briefly, mouse heart and lungs were perfused with 10-15 ml of PBS to remove red blood cells. Lung samples were immediately fixed with 10% formalin for 2 hours at room temperature and stored in 70% ethanol. The tissues were then processed, embedded in paraffin and sectioned. Next, the slides were deparaffinized and stained with Hematoxylin and Eosin (H&E) or Periodic acid Schiff (PAS). Immunohistochemistry: After deparaffinization, slides were washed with PBS and immersed in a solution of methanol and 0.3% H_2_O_2 _for 30 minutes to deplete endogenous peroxidase activity. Slides were stained with 1:100 dilution of rat anti-CD3 (Serotec, Raleigh, NC) followed by 1:200 dilution of biotinylated anti-rat mouse adsorbed antibodies (Vector Laboratories Inc. Burlingame, CA). For FIZZ1 and YM1 staining, sections were incubated first with 10% goat serum for 20 minutes, then stained with a 1:100 dilution of rabbit anti-mouse FIZZ1 (Abcam, Cambridge, MA) or 1:100 dilution of rabbit anti-mouse YM1 (Stem Cell Technologies, Vancouver, Canada) followed by biotinylated anti-rabbit antibodies (1:200; Vector Laboratories Inc.). Finally, all slides were incubated with ABC Elite reagent (Vector Laboratories), developed in 3,3-diaminobenzidine chromogen and counterstained with Mayer's Hematoxylin.

### Assessment of Airway Remodeling

Paraffin embedded lung sections were stained with Masson's Trichrome stain to visualize collagen deposition (blue). Keratin and muscle fibers appear red. To quantify collagen, photomicrographs of Masson's Trichrome stained lung sections (10× and 100× magnification) were obtained and evaluated using NIH Image J image analysis software [46] (National Institutes of Health, Bethesda, MD). Twenty airways/blood vessels were analyzed from each group of mice. Airway smooth muscle thickness was measured using H&E stained lung sections as described in [45]. Briefly, photomicrographs of 100× magnification H&E stained lung sections were taken. The thickness of the airway smooth muscle layer (transverse section) from the innermost aspect to the outermost aspect was measured at 3 different positions around each airway, using NIH Image J software. An average of 30 airways was used for each group.

### Western blot and densitometry analysis

Total protein present in BAL fluid samples isolated from mice was quantified using a micro BCA protein assay kit (Pierce, Rockford, IL). Equal amounts of total BAL protein was run on a 10% or 18% polyacrylamide gel and transferred onto an Immobilon membrane (Millipore, Billerica, MA). The blots were blocked and incubated with antibodies for YM1 (StemCell Technologies) or FIZZ1 (Alpha Diagnostic International Inc., San Antonio, Texas). An anti-rabbit horse radish peroxidase-linked secondary antibody was used (GE Healthcare). Protein bands were detected using a chemiluminescence reagent (ECL; Denville Scientific Inc., Metuchen, NJ). For densitometry analysis, shorter exposure films were scanned and the density of each band was analyzed with the NIH Image software.

### Cytokine and chemokine analysis

Cytokines and chemokines in the BAL fluid were analyzed by using the SearchLight Multiplex array system (Pierce Thermo Scientific; now sold by Aushon Biosystems). IL-4 and IL-5 secretion by splenocytes from naïve or OVA/Alum immunized D011.10xRAG2^-/- ^mice were measured using kits (Pierce Thermo Scientific, Rockford, IL; R&D Systems, Minneapolis, MN) as mentioned in [69]. Briefly, splenocytes were isolated from mice mentioned above and cultured in complete RPMI medium supplemented with 20 U/ml of IL-2 (R&D Systems, Minneapolis, MN) in the presence or absence of PMA (20 ng/ml) (Sigma-Aldrich, St. Louis, MO) and Ionomycin (1 μg/ml) (Sigma-Aldrich) for 18 h. Cell culture supernatants were used to measure cytokine levels.

### FACS analysis

Single cell suspensions of splenocytes were stained with fluorochrome conjugated antibodies and analyzed by flow cytometry using a FACS Calibur machine (Becton Dickinson, Franklin Lakes, NJ). The following antibodies were used for cell surface staining: anti-CD4-PE (clone GK1.5; BD Biosciences), anti-CD4-Alexa Fluor 647 (clone RM4-5; BD Biosciences), anti-DO11.10 TCR-FITC (clone KJ126; eBioscience) and anti-CD44-PerCP Cy5.5 (clone IM7; eBioscience). Intra-cellular cytokine staining was performed to detect IL-4 production. Cells were fixed and permeabilized, followed by staining with anti-IL-4-PE (clone BVD4; BD Biosciences). Data was analyzed by using FlowJo software (Treestar, CostaMesa, CA).

### Statistical Analysis

Student two-tailed *t *test was used to compare the differences between two groups and to calculate the significance values. P values of ≤ 0.05 were considered statistically significant.

## Authors' contributions

PD performed the asthma studies in mice, immunoassays and molecular biology experiments, analyzed data and drafted the manuscript. SCP helped setup the asthma protocol. EPS prepared the lung histology sections and did the immunohistochemical staining. ADK conceived and designed the study, and helped to draft the manuscript. All authors read and approved the final manuscript.

## Supplementary Material

Additional file 1 **Figure S1: Lung inflammation in response to allergen priming and challenge in RAG2^-/- ^and STAT6 or IL-4Rα deficient mice**. *In vivo *primed DO11.10+ CD4+ T cells were adoptively transferred into RAG2^-/-^, STAT6xRAG2^-/- ^or IL-4RαxRAG2^-/- ^mice. Mice were primed with 100 μg of Ova in alum i.p on d. 1 & 6 and then challenged with 1% Ova in PBS on d. 12 &14. Mice were sacrificed, BAL and lung tissue was collected 48 h after the last challenge. (A) Percentages of eosinophils present in the BAL in alum or OVA/alum treated mice are shown. (B) H&E (panels a, d & g- 10X; panels b, e & h- 100X) and PAS (panels c, f & i- 10X) stained lung sections of mice mentioned above. Arrows point areas of inflammation. Data is representative of three independent experiments. Click here for file

Additional file 2 **Figure S2: Absence of STAT6 and IL-4Rα causes reduced eosinophil accumulation in the lung**. Lung sections of Ova primed and challenged mice mentioned in Figure S1 were stained with H&E. Eosinophils and mononuclear cells in each lung section was counted and graphed. Number of cells around the airways (A), blood vessels (B) and in the lung parenchyma (C) are shown. White bars represent eosinophils, black bars represent mononuclear cells. Data represented as cell counts ± SEM. HPF: high power field; 100X. * p < 0.05; + p < 0.01; ** p < 0.0001. # (p < 0.001) represents statistically significant differences when compared to the RAG2KO group. n = 5 for each mouse strain. Click here for file
