# Supplementary figures and images for: Transfer of in vivo primed transgenic T cells supports allergic lung inflammation and FIZZ1 and Ym1 production in an IL-4Rα and STAT6 dependent manner
Source: BMC Immunol. 2011 Oct 20;12:60. doi: 10.1186/1471-2172-12-60 (PMC3212823; doi:10.1186/1471-2172-12-60)

**A.**

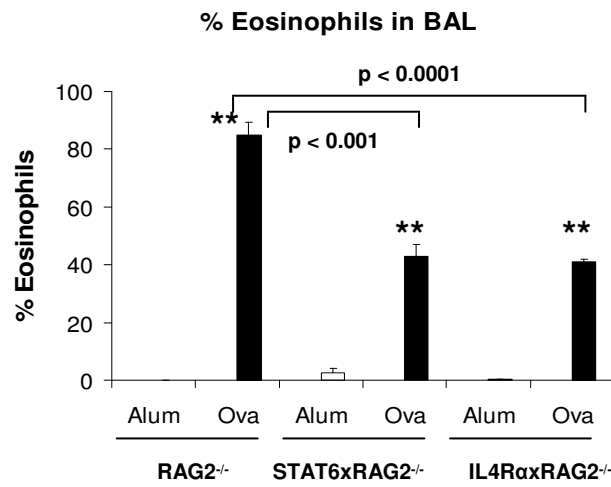

**B.**

Mice:  
+ primed T cells  
+OVA

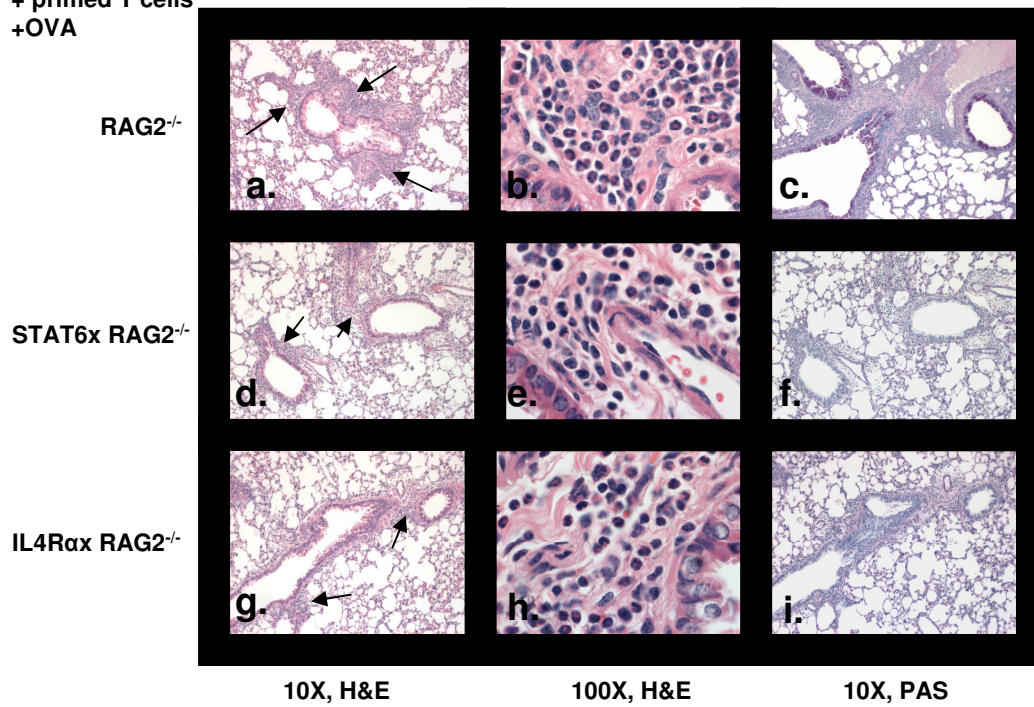

Supplement: Additional file 1 — Figure S1: Lung inflammation in response to allergen priming and challenge in RAG2-/- and STAT6 or IL-4Rα deficient mice. In vivo primed DO11.10+ CD4+ T cells were adoptively transferred into RAG2-/-, STAT6xRAG2-/- or IL-4RαxRAG2-/- mice. Mice were primed with 100 μg of Ova in alum i.p on d. 1 & 6 and then challenged with 1% Ova in PBS on d. 12 &14. Mice were sacrificed, BAL and lung tissue was collected 48 h after the last challenge. (A) Percentages of eosinophils present in the BAL in alum or OVA/alum treated mice are shown. (B) H&E (panels a, d & g- 10X; panels b, e & h- 100X) and PAS (panels c, f & i- 10X) stained lung sections of mice mentioned above. Arrows point areas of inflammation. Data is representative of three independent experiments. [file 1471-2172-12-60-S1.PDF]

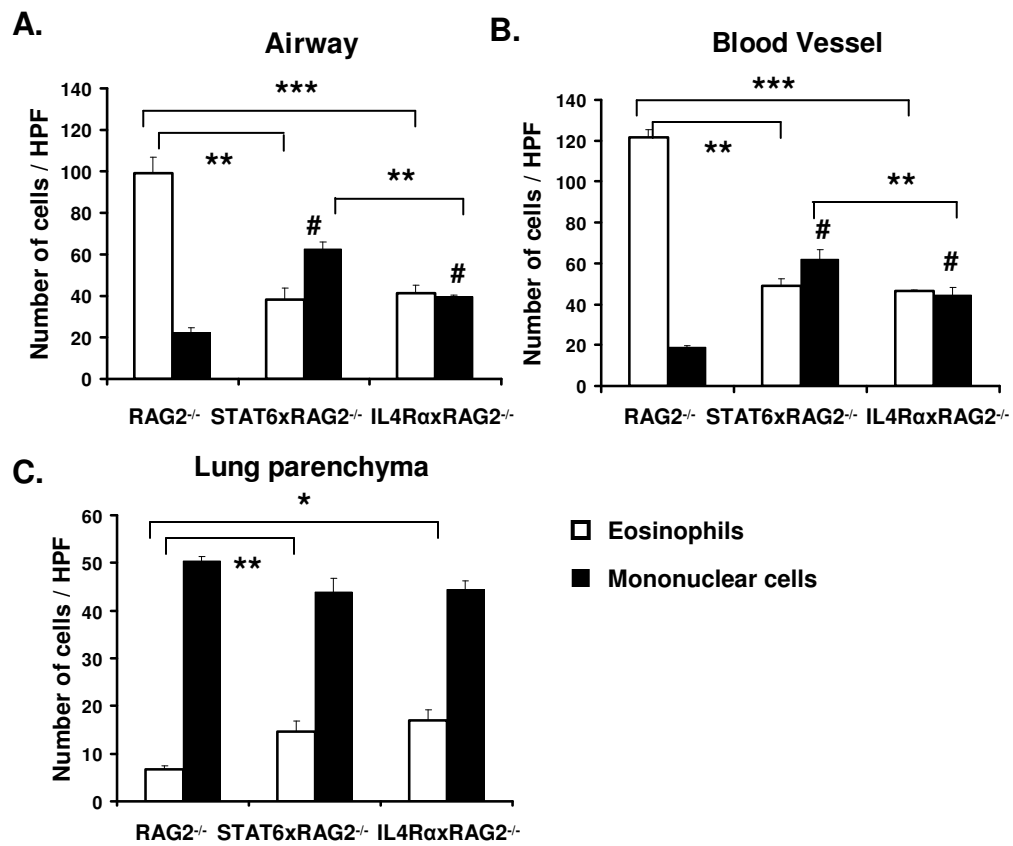

Supplement: Additional file 2 — Figure S2: Absence of STAT6 and IL-4Rα causes reduced eosinophil accumulation in the lung. Lung sections of Ova primed and challenged mice mentioned in Figure S1 were stained with H&E. Eosinophils and mononuclear cells in each lung section was counted and graphed. Number of cells around the airways (A), blood vessels (B) and in the lung parenchyma (C) are shown. White bars represent eosinophils, black bars represent mononuclear cells. Data represented as cell counts ± SEM. HPF: high power field; 100X. * p < 0.05; + p < 0.01; ** p < 0.0001. # (p < 0.001) represents statistically significant differences when compared to the RAG2KO group. n = 5 for each mouse strain. [file 1471-2172-12-60-S2.PDF]
